# Amplification of vibration induced nystagmus in patients with peripheral vestibular loss by head tilt

**DOI:** 10.3389/fneur.2024.1420699

**Published:** 2024-10-16

**Authors:** Ari Aharon Shemesh, Jorge C. Kattah, David S. Zee, Francisco Zuma E Maia, Jorge Otero-Millan

**Affiliations:** ^1^The Technion Autonomous Systems Program, Technion – Israel Institute of Technology, Haifa, Israel; ^2^Department of Neurology, University of Illinois College of Medicine, Peoria, IL, United States; ^3^Department of Neurology, The Johns Hopkins University, Baltimore, MD, United States; ^4^Department of Otolaryngology-Head and Neck Surgery, The Johns Hopkins School of Medicine, Baltimore, MD, United States; ^5^Department of Ophthalmology, The Johns Hopkins School of Medicine, Baltimore, MD, United States; ^6^Department of Neuroscience, The Johns Hopkins School of Medicine, Baltimore, MD, United States; ^7^Department of Otorhinolaryngology and Instituto de Cerebro, Pontifical Catholic University of Rio Grande do Sul, Porto Alegre, Brazil; ^8^Herbert Wertheim School of Optometry and Vision Science, University of California, Berkeley, Berkeley, CA, United States

**Keywords:** nystagmus, vestibular testing, vestibular neuritis, gravity estimation, three dimensions

## Abstract

**Introduction:**

In patients with unilateral loss of vestibular function (UVL) vibration of the skull leads to a response of the vestibulo-ocular reflex (VOR) called vibration-induced nystagmus (VIN), with slow phases usually directed toward the paretic ear. This response is thought to result from the difference between the neural discharge in semicircular canal afferents from the healthy and the affected labyrinth. The brain interprets this difference as a sustained imbalance in angular (rotational) vestibular tone, which in natural circumstances would only occur when the head was rotating at a constant *acceleration*.

**Methods:**

To study this effect, we used a contemporary model of the neural network that combines sensory information about head rotation, translation, and tilt relative to gravity to estimate head orientation and motion. Based on the model we hypothesize that in patients with UVL, the brain may estimate not only a “virtual” rotation from the induced canal imbalance but also a subsequent “virtual” translation from the incorrect computation of the orientation of the head relative to gravity. If this is the case, the pattern of vibration-induced nystagmus will depend on the orientation of the head relative to gravity during the stimulation. This model predicts that this “virtual” translation will alter the baseline VIN elicited with the head upright; augmenting it when the affected ear is down and diminishing it when the affected ear is up.

**Results:**

Confirming this hypothesis, we recorded VIN in 3 patients with UVL (due to vestibular neuritis) in upright, right ear-down, and left ear-down positions and each showed the expected pattern.

**Discussion:**

From a practical, clinical view, our results and modeling suggest that positional VIN might reveal a hidden imbalance in angular vestibular tone in patients with UVL, when patients have equivocal signs of a vestibular imbalance, such as a minute amount of spontaneous or vibration-induced nystagmus with the head upright. This research provides insights into the underlying mechanisms of vestibular processing, the analysis of nystagmus in patients with UVL, and guides the design of a new bedside diagnostic test to assess vestibular function in patients with dizziness and imbalance.

## 1 Introduction

Vibration of the skull is used as clinical bedside test to detect an imbalance in the vestibular system, typically eliciting a vibration-induced nystagmus (VIN) in patients with a unilateral loss of vestibular function, for example, due to vestibular neuritis (1). Here we propose a new way to use the effect of vibration of the skull in patients that increases its sensitivity for detecting an imbalance in vestibular tone, and furthermore, is a test of current models of how the brain computes the orientation of the head with respect to gravity. In the introduction we first provide the theoretical background and describe a three-dimensional model for how the direction of gravity is estimated by the brain, and then discuss how vibration of the skull in patients with unilateral vestibular loss might affect this computation.

### 1.1 Virtual head rotations and virtual translations interact with estimates of the orientation of the head with respect to gravity

In patients with unilateral loss of vestibular function, such as due to vestibular neuritis, the brain receives a constant imbalance in angular (rotational) vestibular tone when the head is stationary. The primary manifestation of this imbalance is a sustained spontaneous nystagmus that is driven by an erroneous interpretation by the brain that the head is rotating with a constant *acceleration* toward the side of the intact ear (2). Accordingly, to accurately interpret spontaneous nystagmus in patients, one must appreciate that the vestibulo-ocular reflex (VOR) can also generate nystagmus in response to virtual, i.e., not actual, head rotations even when the head is still. For example, even in normal subjects with the head still, a false compensatory nystagmus is produced from a temporary imbalance in peripheral labyrinthine activity that occurs when the head stops moving after a sustained rotation (post-rotatory nystagmus), or with caloric irrigations. Likewise, a false compensatory nystagmus can be produced when there is an imbalance in central vestibular tone, such as the positional nystagmus shown by patients who have neurological disorders involving the cerebellum or brainstem (3–7). Understanding how the brain computes the orientation of the head – from a real or a virtual head motion – can help explain the changes in patterns of spontaneous nystagmus and in associated symptoms that occur after changes in head position in both normal subjects and in patients (7, 8).

### 1.2 A three-dimensional model for generating vestibular nystagmus

Head rotations, real or virtual, have an axis of rotation that is an imaginary line around which the head revolves. When this axis is aligned with the direction of gravity, the orientation of the head relative to gravity is unaffected by the rotation. However, when a patient with a peripheral vestibular imbalance, for example with an acute unilateral vestibular neuritis, assumes a static position in which the axis of virtual head rotation is not aligned with the direction of gravity, the brain will update its estimate of the direction of gravity as though the virtual head rotation were real. Other examples include artificially induced nystagmus (e.g., with a caloric or vibration stimulus, or post rotatory nystagmus) in which case the head is still but nevertheless is interpreted as a real rotation of the head. As a result, the estimate of the orientation of the head is based on a fusion of the inputs from the semicircular canals, which are falsely interpreted as a veridical head rotation, and the static signals from the otolith organs, which indicate that the orientation of the head with respect to gravity is unchanging. This will produce a *misalignment* between the direction of gravity estimated centrally and the direction of the GIA (gravito-inertial acceleration) sensed by the otolith organs.

**Figure 1:**
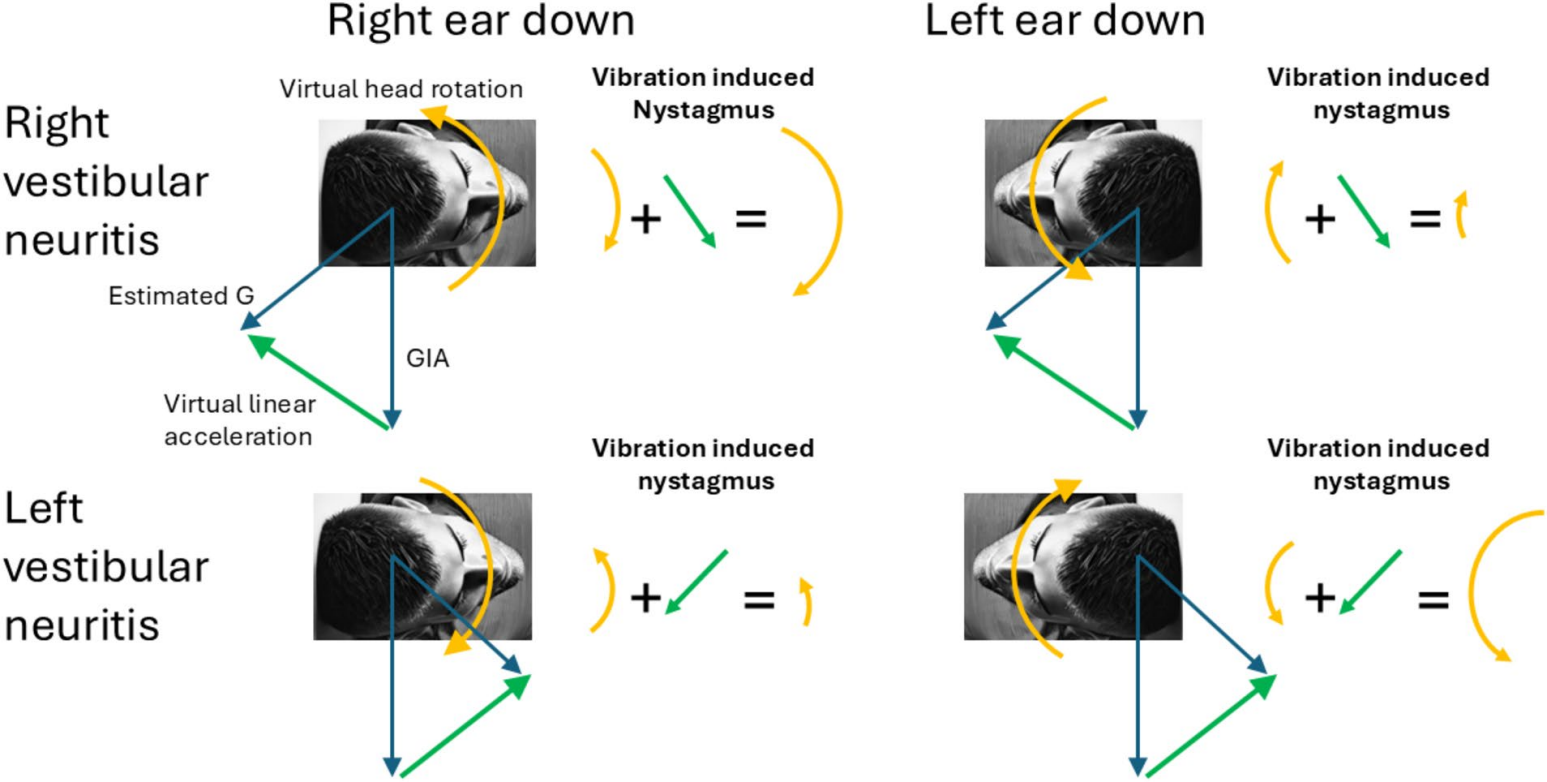
Virtual rotations and virtual translations. The figure shows how under different lesions and different head tilts the different linear and rotational components add to produce the observed pattern of vibration induced nystagmus. Notably, in ear-down positions, the erroneous estimated gravity (G) shifts forward or backward along the sagittal plane, rising above the GIA. Consequently, the interaural virtual linear acceleration always points upward toward the upper ear (green arrow). The arrows representing the direction of the nystagmus correspond with the direction of the slow-phases produced by the corresponding virtual rotations and virtual linear accelerations.

The GIA reflects the combined linear accelerations from the pull of gravity and from any translation of the head. The brain will attribute a discrepancy between the GIA and the central estimate of the direction of gravity to a virtual *linear acceleration* (or translation) of the head which in turn leads to another false compensatory response, by the translational VOR (t-VOR), adding to the nystagmus of the patient (Figure 1). The positioning of the head can either augment or decrease the spontaneous or artificially-induced nystagmus depending on the interaction between the virtual rotational and the virtual translational VOR. This interaction is well depicted in Merfeld et al. (9), e.g., Figure 4A. For example, in the acute clinical setting, patients with an acute unilateral vestibular loss due to vestibular neuritis, often attempt to minimize their symptoms by preferring to lie on their side with the affected ear up (10). In this position, the virtual translational VOR lessens the spontaneous horizontal nystagmus arising from the imbalance in the angular VOR, while lying with the healthy ear up, the virtual translational VOR enhances the spontaneous nystagmus.

**Figure 2:**
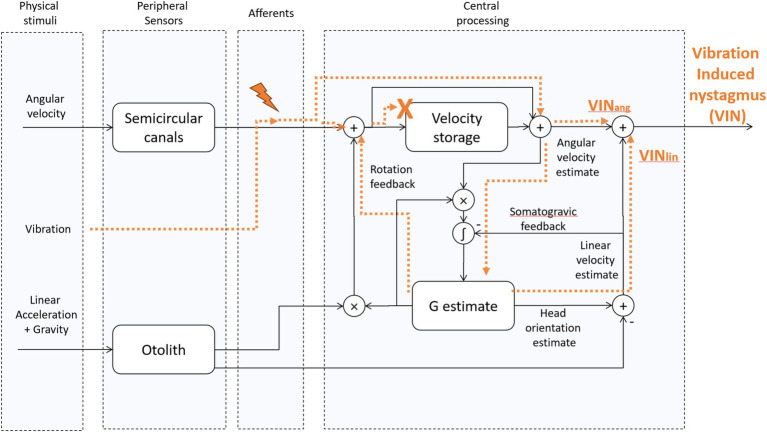
Tilt translation ambiguity 3D Model [adapted from “Modeling the effect of gravity on periodic alternating nystagmus,” (7)]. The vibration stimulus affects the afferents of the SCCs. Then, their activity is centrally processed as a virtual rotation, and it also leads to a virtual translation whenever the virtual rotation axis is not aligned with gravity. This occurs, for example, when one ear is in a downward position with the head titled away from upright. We hypothesize that because of the unnatural characteristics of the vibration-induced stimulus, i.e., its activity is not fed back through the velocity-storage, the rotation feedback loop can no longer influence the VIN. Likewise, we hypothesize that because of the multidirectional orientations of the otolith hair cells on the macula, the artificial nature of any stimulation of otolith afferents does not give a coherent signal to the brain about the GIA. VINang = the angular component of VIN, VINlin – the linear component of VIN. “X” indicates that VIN does not have access to the velocity storage. The lightning symbol indicates vibration-induced activation of type 1 (irregular) semicircular canal afferents.

Computational models help us understand how the brain normally resolves a tilt-translation ambiguity and correctly extracts the two component parts of the GIA signal transduced by the otoliths: tilt relative to gravity and linear acceleration (9–14). At the core of these models is a velocity-storage mechanism, aided by two feedback loops—the rotation feedback and the somatogravic feedback loops (Figure 2)—which help keep the central estimate of gravity aligned with the GIA from the otolith organs, which is the natural expected state for sustained linear acceleration. The *rotation feedback loop* modulates activity in the velocity-storage system to correct conflicting estimates of angular velocity, often arising from aftereffects of stimulation of the canals, that would otherwise be mathematically integrated into incorrect tilt estimates. As an example, the suppression of unwanted nystagmus when the head has just stopped rotating around an earth vertical axis and also titled away from upright is a result of the rotation feedback loop (11). The *somatogravic feedback loop* acts over a longer time scale by introducing a low-pass filtered version of the GIA to the central gravity estimator (G estimate, Figure 2) to interpret sustained forces as tilts relative to gravity and not constant translations. This feedback loop also helps to eliminate any disparity between the GIA and estimated gravity, ensuring both realignment and matching of their magnitudes (15–21).

### 1.3 Nystagmus induced by vibration of the skull (VIN)

The response to a vibration stimulus applied to one mastoid is propagated throughout the skull, exciting the vestibular receptors in both labyrinths. This initiates a phase-locked activation of the irregular (type 1) afferents from both labyrinths (22) (Figure 2). Typically, healthy individuals do not exhibit vibration-induced nystagmus (VIN) since excitation of both labyrinths is balanced. In patients with unilateral vestibular loss, however, the heightened neural firing from the healthy labyrinth does not get neutralized by activity from the other labyrinth, and leads to a primarily horizontal nystagmus with quick phases usually directed away from the affected side (23–27).

The virtual rotation induced by the vibration stimulus differs from natural stimulations in at least two important aspects. First, the neural discharge is time-locked to the vibration stimulus because afferents from the labyrinth are activated at a later stage, independently of the mechanics of the cupula and endolymph within the semicircular canals. Second, as mentioned above, there may be a different relative activation of irregular and regular afferents. We hypothesize that because of those differences, the response to the vibration-induced virtual rotation uniquely bypasses the central vestibular velocity-storage. This is consistent with the fact that VIN stops immediately when the stimulus is removed (22), implying the stimulus does not activate the velocity-storage. As the effects of rotation feedback are mediated through velocity-storage, which is not engaged during VIN, the rotation feedback can no longer help solve the tilt-translation ambiguity, leaving this task solely to the somatogravic feedback loop.

Although VIN is typically tested with the patient upright, vibration to the skull can also be applied with patients lying in ear down positions. We hypothesize that by changing the orientation of the head relative to gravity one can use the vibration-induced response to manipulate the direction and strength of the translational VOR. In turn, this response can help identify patients who have suffered a vestibular disorder, but only have a hint of a VIN with the head upright or show little or no spontaneous or head-shaking induced nystagmus, as other signs of a vestibular imbalance. By taking advantage of the effects of the translational VOR in this way, clinicians may uncover a vestibular tone imbalance at the bedside that might otherwise have gone unnoticed.

## 2 Methods

We recruited patients at the neuro-otology clinic within the OSF (Sisters of the Third Order of St. Francis) Illinois Neurological Institute with a confirmed history of peripheral vestibular neuritis, including both subacute and chronic cases. Since a goal of our study was to develop a method for identifying a vestibular tone imbalance in patient with tiny or no spontaneous nystagmus, we excluded those with a spontaneous nystagmus when visual fixation was removed and when following head-shaking, of more than 2 deg/s.

Eye movements were recorded with fixation removed, and nystagmus analyzed using the ICS CHARTR 200 goggles (Natus Medical Incorporated, Denmark). A vibration stimulus at 100 Hz (Homedics) was applied to the mastoids for 1 min to elicit vibration-induced nystagmus in three head orientations; upright, right ear-down and left ear-down. Slow-phase velocity was calculated as the average slow velocity during the stimulation. The device features a switch located at the apex of its triangular shape, which includes three bulges that comfortably fit over the mastoid process, matching its size.

In the ear-down tests, patients were initially tilted to the ear-down position en-bloc, and then tested for vibration-induced nystagmus. Patients were told to look straight ahead. In the upright position, stimulation of either the ipsilesional or contralesional mastoid produced no differential responses, highlighting that the vibration stimulus is transmitted effectively to both labyrinths, regardless of which side is stimulated (22). In the ear-down positions, the vibration stimulus was placed on the mastoid opposite to the side of the head that was down—on the left mastoid when the right ear was down and vice versa for the left ear down.

Three patients who met the criteria were included in the study (Table 1). The first was examined 3 weeks after a diagnosis of right vestibular neuritis. The second patient was evaluated 1 year after a left vestibular neuritis and the third two years after a right vestibular neuritis. In the absence of a vibration stimulus, only the first patient showed a spontaneous nystagmus, with a speed of 2 degrees/s with the head upright, which increased to 3 deg/s with the right (affected) ear down. The other two patients did not show any spontaneous nystagmus or positional nystagmus with either ear down. Additionally, three control subjects were also recorded without any nystagmus present with vibration stimulation in either head position.

**Table 1:** Patient information.

	Diagnosis	Age	Gender race	Date of AVS onset	Recording VIN	Initial video head impulse	Bithermal calorics	Sinusoidal harmonic oscillation	Post head shaking nystagmus
1	Right Superior/ Inferior Vestibular Neuritis	73	M.A.A	09–13-22	10–07-22	LL 0.71 RL:0.42 LA:0.88 RP:0.30 RA:0.76 LP:0.69	37% R Canal paresis	Not done	Not present
2	Right Superior/ Inferior vestibular Neuritis	56	F.C.	11–15-20	11–23-22	LL: 0.91 RL:0.32 LA:0.58 RP:0.45 RA:0.50 LP:0.83	65% R Canal paresis	Not done	Not present
3	Left Superior/Inferior Vestibular Neuritis ^*^	57	M.C	06–30-22	11–23-23	LL: 0.15 RL: 0.34 LA:0.34 RP:0.84 LP:0.75 RA: 0.99	100% L Canal paresis	Low gain at all frequencies. No phase lead. Counterclockwise Asymmetry.	Present, h-RBN, SPV 2 deg/s for >1 min

* The patient had absent left cVEMP and oVEMP.

Additional tests were also performed, such as head impulse of the 6 canals and bithermal calorics. Results are included in Table 2.

**Table 2:** Nystagmus characteristics.

	A	B	C	D	E	F	G
Patient	Affected vestibular neuritis side	Duration since symptoms onset	Vibration sitting with head upright	Vibration in RED	Vibration in LED	Estimated angular VOR contribution to vibration induced nystagmus	Estimated linear VOR contribution to vibration induced nystagmus (RED, LED^*^)
1	Right	3 weeks	6 d/s	8 d/s	2.5 d/s	5.25 d/s	+ − 2.75 d/s
2	Right	2 years	5.1 d/s	10 d/s	0 d/s	5 d/s	+ − 5 d/s
3	Left	1.5 years	−14.1 d/s	−7 d/s	−20.6 d/s	−13.8 d/s	+ − 6.8 d/s

* Right ear down (RED), left ear down (LED). Positive numbers indicate slow-phase velocity toward the right.

## 3 Results

### 3.1 Effect of head position on vibration induced nystagmus (VIN)

**Figure 3:**
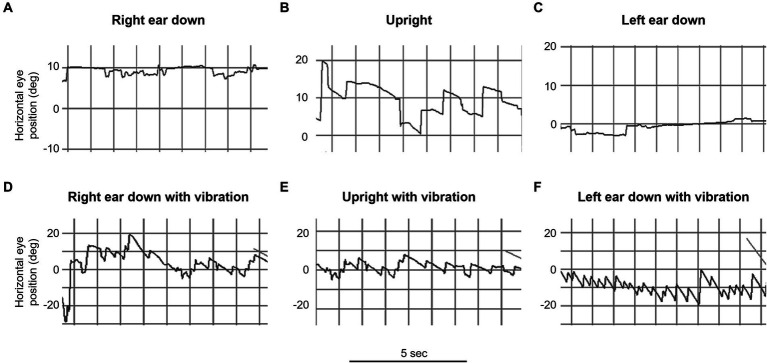
Video oculography (VOG) of mastoid vibration in patient 3 (left vestibular neuritis). This figure illustrates horizontal eye movement recordings with fixation removal under various positions over 5-s intervals. The upper row represents positional testing *without* vibration: **(A)** right ear down, **(B)** upright, and **(C)** left ear down. The lower row represents positional testing *with* vibration: **(D)** right ear down, **(E)** upright, and **(F)** left ear down. Note the marked change in the intensity of the vibration induced nystagmus with changes in the head orientation relative to gravity (e.g., bottom row, compare **D** and **F**).

Our main result shows that our clinical findings confirm the predictions of our model (Table 2 and Figure 3). When patients were positioned with their “bad” ear down, the compensatory translational VOR added to the spontaneous nystagmus associated with the angular imbalance. This resulted in a higher speed VIN compared to when the patients were upright. On the other hand, when patients were positioned with their “good” ear down, the translational VOR subtracted from the spontaneous nystagmus associated with the angular imbalance. This led to a lower velocity VIN compared to the seated position (Table 2, columns C, D, and E).

Considering that the translational VOR can either augment or diminish the spontaneous nystagmus associated with angular imbalance, it is feasible to determine the contribution of the translational VOR quantitatively (28, 29). Table 2 shows the result of these calculations for our patients and we show here a detailed example for one hypothetical case.

Let us consider a hypothetical example of right vestibular neuritis. In the right ear-down position, the total observed $VIN_{R}$ is 15 deg/s. According to our model, this corresponds to the sum of the angular and linear components $VIN_{R}=VIN_{ang}+VIN_{lin}$ . In the left ear-down position, the total observed $VIN_{L}$ is 5 deg/s. Here, their corresponds to $VIN_{L}=VIN_{ang}-VIN_{lin}$ . To isolate the angular component, $VIN_{ang}$ , we can average the total VIN across both ear-down positions because:


$$\frac{VIN_{R}+VIN_{L}}{2}=\frac{\left(VIN_{ang}+VIN_{lin}\right)+\left(VIN_{ang}-VIN_{lin}\right)}{2}=VIN_{ang}$$

By inserting our observed values, we get:


$$VIN_{ang}=\frac{15+5}{2}=10$$

This result indicates that the angular imbalance component, $VIN_{ang}$ , is 10 deg/s.

Next, to determine the linear imbalance component, Lin, we can subtract the VIN values observed in each ear-down position from one another and divide the result by 2. The formula is:


$$\frac{VIN_{R}-VIN_{L}}{2}=\frac{\left(VIN_{ang}+VIN_{lin}\right)-\left(VIN_{ang}-VIN_{lin}\right)}{2}=VIN_{lin}$$

Again, using our observed values, we find:


$$VIN_{lin}=\frac{15-5}{2}=5$$

Thus, the contribution of the linear imbalance component, Lin, is 5 deg/s.

Note that in these calculations we do not consider a potential effect of the rotation feedback modulating the response from the VIN when the head orientation changes, as would be the case when the head is tilted in the setting of a peripheral vestibular imbalance. The exclusion of an effect from rotational feedback and velocity storage is based on the results of previous studies (22) in which there is no post vibration induced nystagmus response. This suggests that the vibration stimulus does not engage the velocity-storage and therefore bypasses the rotation feedback loop. We expect the component of the VIN in ear-down positions from the angular imbalance to be close to the VIN observed when the head is upright, a position in which no imbalance in linear vestibular tone is to be expected. Thus, the vibration induced imbalance in angular vestibular tone should remain the same across these head positions, and this is the case (Table 2, columns C and F). Note that the VIN in the head upright positions was not included in the calculations for the estimated angular component.

## 4 Discussion

### 4.1 Clinical implications of virtual rotations and translations in patients with unilateral vestibular hypofunction

In this study, we showed that positional vibration-induced nystagmus can be used as a bedside test to reveal a hidden angular vestibular tone imbalance. This imbalance can go unnoticed in subacute and chronic settings when bedside assessments rely primarily on spontaneous and head-shaking nystagmus, which may not be present or so small as not to be considered pathological. Only a minimal or no spontaneous nystagmus in these patients can be the result of set-point adaptation, the system that identifies the new resting balance and eliminates the unwanted spontaneous nystagmus response (30). However, an imbalance in the dynamic response, as reflected in the asymmetrical gain response of the VOR to head impulses, may still be present. In fact, head-impulse testing in these patients showed this to be the case (Table 2).

Using a 3D model for how the brain normally estimates the orientation of the head relative to gravity, we first considered the finding that VIN immediately stops when the stimulus is removed. We then inferred that velocity-storage was not engaged by the vibration stimulus as discussed above, there is either no after effects associated with VIN or only rarely, a very low-velocity sustained nystagmus that is likely due to a nonspecific excitation (or arousal) in central activity (22). Under this assumption, there are no effects of the rotation feedback loop on VIN and only the somatogravic feedback can have a gravity-dependent influence on VIN by interpreting the sustained linear acceleration as tilt relative to gravity rather than as constant translation, thereby affecting the linear (translational) VOR contribution. This is particularly relevant when patients with a persistent angular tone imbalance, such as those with unilateral vestibular loss, adopt an ear-down position. This leads to a sustained misalignment of the axis of virtual head rotation with gravity since the somatogravic feedback is not strong enough to completely counteract the effect of the continuous virtual rotation. Any residual misalignment between the GIA and the estimated gravity is then interpreted as a constant virtual head translation (31). Therefore, in these patients, eliciting vibration-induced nystagmus in ear-down positions adds or subtracts a constant nystagmus that compensates for the inferred virtual head translation. Thus, the changes in VIN induced by vibrating the mastoid in different head orientations can enhance the detectability of a vestibular tone imbalance. As we show in Table 2, the results of VIN are consistent with the results of the head-impulse test, but a mild imbalance might be easier to detect with VIN than with HIT. Indeed, this is often the challenge when trying to find a cause in patients with mild complaints of imbalance or dizziness. The nystagmus may even be enhanced at other head orientations depending on how much the estimated gravity direction has shifted (Figure 1). The optimal head orientation would align the head toward and against the estimated head orientation for maximal effect of the virtual linear acceleration. However, it would be hard to know *a priori* how to exactly orient the patient and a similar result is obtained with left and right ear down positions.

### 4.2 The somatogravic feedback loop

The somatogravic feedback functions as a low-pass filter for the GIA output of the otolith, and its behavior can be characterized by its time constant. As demonstrated by Glasauer et al. in patients with unilateral vestibular loss in a static body position, the time constant of the somatogravic feedback governs the steady-state estimated gravity and, consequently, the virtual linear acceleration, which represents the misalignment between the GIA and estimated gravity (31). Consequently, a lower somatogravic time constant corresponds to a weaker linear VOR, while a higher time constant leads to a stronger linear VOR. This suggests that patients with the same level of angular vestibular tone imbalance may exhibit varying intensities of nystagmus during the vibration positional test, depending on their respective somatogravic time constants. Previous studies have documented somatogravic time constants spanning from 1.5 to 7 s, supporting the plausibility of this scenario (32–37).

### 4.3 Differences between head shaking and vibration induced nystagmus

Head-shaking nystagmus and vibration nystagmus have different characteristics and methods of evaluation (38). The head-shaking nystagmus test assesses angular vestibular tone imbalance by inducing asymmetric “charging” of the right and left velocity-storage circuits differently during head shaking. This can be understood from Ewald’s Second law, which states that excitation is a more effective stimulus than inhibition. The resulting nystagmus after the head stops moving, reflects the asymmetrical “discharge” of the stored velocity. In contrast, vibration nystagmus occurs during the vibration stimulation itself and does not involve the activation of the velocity-storage (22). The explanation for this bypass is uncertain but could be related to the differential activation and timing of type 1 and type 2 afferents. When evaluating head-shaking induced nystagmus, it is clinically helpful to incorporate head tilting after the head shaking has taken place since an absence of tilt suppression of head shaking induced nystagmus may signal a central disorder, in the same way as an absence of tilt suppression of post rotatory nystagmus (39–44). This is because the effect of the rotation feedback loop, mediated through the velocity-storage, accelerates the decay of the inappropriate nystagmus induced following head shaking. In contrast, vibration with head tilting should not yield the same results to head shaking as it does not engage the rotation feedback. Consequently, positional VIN may not be able to provide the same discriminatory value in distinguishing between peripheral and central vestibular causes, but nevertheless it can be a reliable and sensitive sign of angular imbalance somewhere in the vestibular system, by making it more noticeable (through a more intense VIN) at the bedside. Furthermore, when investigating nystagmus induced by head-shaking in ear-down positions, one would not anticipate the same positional effects as seen in VIN. This is because the presence of rotation feedback can nullify the contribution of the linear VOR by aligning the estimated gravity with the GIA. Consequently, the influence of the linear VOR during head shaking in ear-down positions could be masked by the effects of rotation feedback on the velocity-storage. Positional VIN is not subject to this masking effect.

### 4.4 “Wrong way” vibration induced nystagmus

Approximately 8% of vestibular neuritis cases have VIN in the head upright position with slow phases directed toward the intact side (45). The tilt-translation model offers an insight into this phenomenon. It suggests that when the axis of virtual rotation does not align with gravity with the head upright in a seated position, there might be a combined effect of virtual rotation and virtual translation. The compensatory translational VOR can augment or diminish the compensatory rotational VOR associated with the angular imbalance. If the translational VOR is strong enough to overcome the rotational VOR, the VIN might exhibit a slow phase directed away from rather than toward, the affected side. In patients experiencing spontaneous vestibular horizontal nystagmus, the clinical implication of the model allows for a nuanced analysis of the imbalance of angular tone in ear-down positions—like methods used for VIN—by presuming that the rotation feedback operates equally in both right and left ear-down orientations. This analysis can then be compared to the nystagmus observed when the patient is in an upright position. A discrepancy between these observations suggests that the nystagmus in an upright position is due to the VOR compensating for both angular and linear imbalances. Future research could explore whether patients in an upright position with nystagmus that compensates for both rotational and translational movements are more susceptible to a convergence stimulus and exhibit asymmetry in their response to rapid translational horizontal head movements (heaves), akin to the head impulse test but for linear rather than angular stimulation (46, 47).

### 4.5 Caveats and limitations of our approach

A potential drawback of our model is that it does not account for the stimulation of otoliths resulting from a 100 Hz vibration stimulus or stimulation of the vertical semicircular canals. It has been shown that stimulation at such a frequency engages both canal and otolith structures in animals, as indicated by experiments involving anaesthetized guinea pigs. During these studies, primary SCC and otolithic afferents reacted to the vibration stimuli (48). Since VIN predominantly shows a horizontal component one can hypothesize that the afferents from the horizontal SCCs are the main target affected by the stimulus. In addition, the diffuse nature and lack of any directionality to the stimulation of the otolith afferents also suggests that the artificial nature of stimulation of otolith afferents cannot give a coherent signal to the brain about the GIA, and therefore this would minimize any potential direct otolith influence on VIN. Finally, VIN has proven to be a useful diagnostic indicator in clinical practice for lesions involving the semicircular canals given its strong correlation (1). Hence, in our model, any “virtual” head translation originates from the brain processing “virtual” rotation signals as actual rotations in its internal calculations and does not depend on any direct stimulation of otolithic afferents. Another caveat is that we cannot exclude some effect of stimulating cervical afferents as vibration of the neck can alter the perception of gravity (49). We did not study the effect of convergence on VIN which may influence the response of the translational VOR. Even with these caveats the essential interpretation of the findings in our patients, and the clinical implications are compatible with current models of how the brain disambiguates tilt and translation.

## 5 Conclusion

The model that has been developed over the past decades to explain how the brain distinguishes between tilt and translation holds important implications for the interpretation and development of new bedside examination techniques. This model provides valuable insights into the underlying mechanisms of vestibular processing and guides the design of diagnostic tests to accurately assess vestibular function. By incorporating this understanding into clinical practice, clinicians can refine and enhance the effectiveness of bedside examinations, resulting in more accurate diagnoses and improved patient care. At the same time these new clinical findings become important tests of our concepts and models of how the brain normally processes sensory information to optimize vision during movement of the head.

## Data Availability

The raw data supporting the conclusions of this article will be made available by the authors. Requests to access the datasets should be directed to jom@berkeley.edu.
